# Genome Sequence of SCB34, a Sequence Type 131 Multidrug-Resistant *Escherichia coli* Isolate Causing Neonatal Early-Onset Sepsis

**DOI:** 10.1128/genomeA.00514-14

**Published:** 2014-06-12

**Authors:** Susana Chavez-Bueno, Michael W. Day, Inimary T. Toby, Darrin R. Akins, David W. Dyer

**Affiliations:** a Department of Pediatrics, Oklahoma University Health Sciences Center, Oklahoma City, Oklahoma, USA; b Department of Microbiology and Immunology, Oklahoma University Health Sciences Center, Oklahoma City, Oklahoma, USA

## Abstract

SCB34 is a sequence type 131, highly invasive, multidrug-resistant *Escherichia coli* isolate that produced neonatal bacteremia. Whole-genome sequencing was performed using a 250-bp library on the Illumina MiSeq platform; 5,910,264 reads were assembled *de novo* using the A5 assembly pipeline. The total contig length was 5,227,742 bp; the RAST server was used for annotation.

## GENOME ANNOUNCEMENT

*Escherichia coli* strains belonging to sequence type (ST) 131 have disseminated worldwide as a cause of severe urinary tract infections and bacteremia (1). *E. coli* is a predominant pathogen causing neonatal sepsis and meningitis, and bacteremia-associated ST131 strains have only recently been reported in newborns (2–4). ST131 strains are commonly multidrug resistant (MDR) and show a high prevalence of extended-spectrum beta-lactamase (ESBL) resistance in some geographic regions (5). SCB34 is the first sequenced ST131 neonatal early-onset sepsis isolate. It was recovered from a 6-day-old premature newborn born at 22 weeks gestation. The mother had peripartum fever and acute chorioamnionitis for which she received ampicillin and gentamicin. Subsequent placenta culture did not yield pathogens. The newborn received ampicillin and amikacin immediately after birth but decompensated on day 6, when a blood culture grew *E. coli*. The local Institutional Review Board approved the collection of the clinical isolate and pertinent clinical data. Antimicrobial susceptibility testing, performed in accordance with CLSI guidelines (6), revealed that while SCB34 is not an ESBL-producing strain, it is resistant to ampicillin, gentamicin, tobramycin, and ciprofloxacin. While human ST131 isolates have shown variable adherence to and invasion of epithelial cells (4, 7), SCB34 has been shown to be highly invasive compared to other ST131 clinical isolates from bacteremic newborns (4).

Whole-genome sequencing was performed on an Illumina MiSeq using a 250-bp paired-end library, which generated 5,910,264 reads with an average read length of 208 bp. The paired-end reads were assembled *de novo* using the A5 assembly pipeline (8). This yielded a database with 80 contigs, for a total contig length of 5,227,742 bp, with a 50.6% G+C content and an *N*_50_ of 205 kb. These data were submitted to the RAST server for annotation (9). Analysis of the *adk*, *fumC*, *gyrB*, *icd*, *mdh*, *purA*, and *recA* alleles confirmed that SCB34 is an ST131 strain (10). Although SCB34 is MDR, we did not find any evidence for plasmids in this strain, but we identified chromosomal antibiotic resistance determinants consistent with the antibiogram of this organism. Additional putative virulence factors common to pathogenic ST131 isolates (11) were identified in the genome, including the pathogen-associated iron transport *iuc* and *fyu* loci, at least three fimbrial gene clusters, several adhesins, and an RTX toxin. Other loci of note included curlin and cellulose biosynthesis loci (12, 13), both of which are important for biofilm formation. These data will be essential to our understanding of the properties of SCB34 contributing to the ability of this organism to cause neonatal bacteremia.

### Nucleotide sequence accession numbers.

This whole-genome draft shotgun sequence has been deposited at GenBank under accession number JMKH00000000. The version described in this report is version JMKH01000000.
